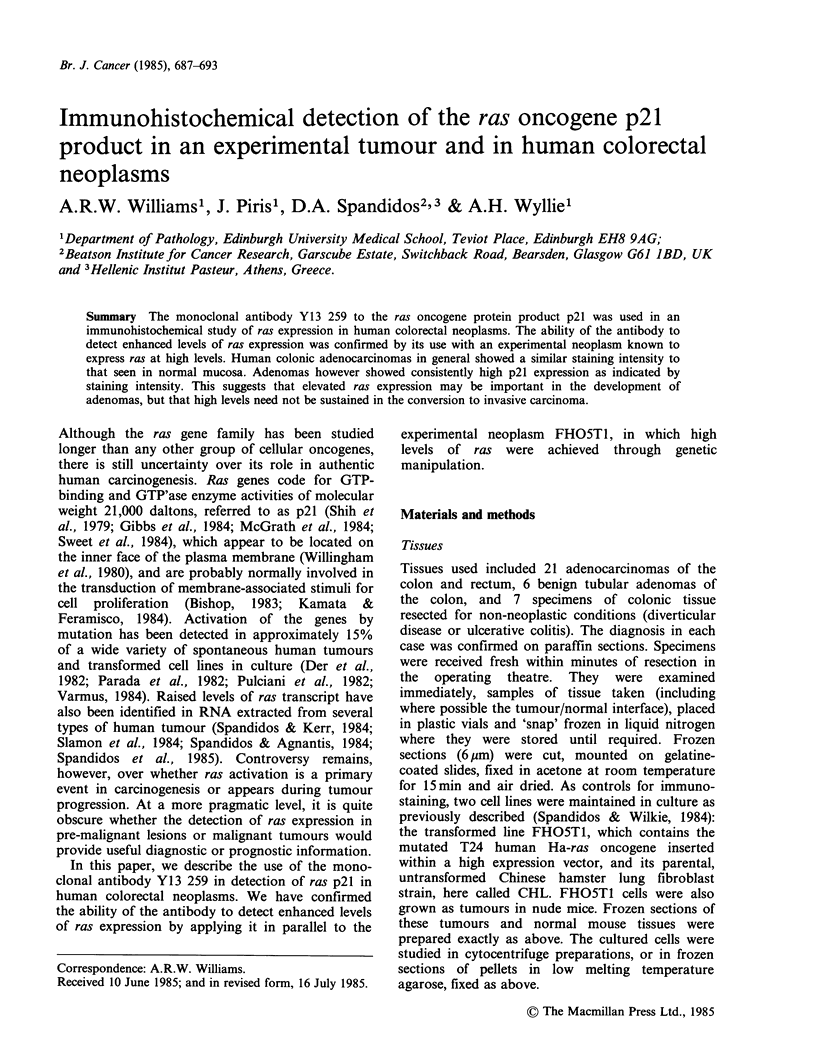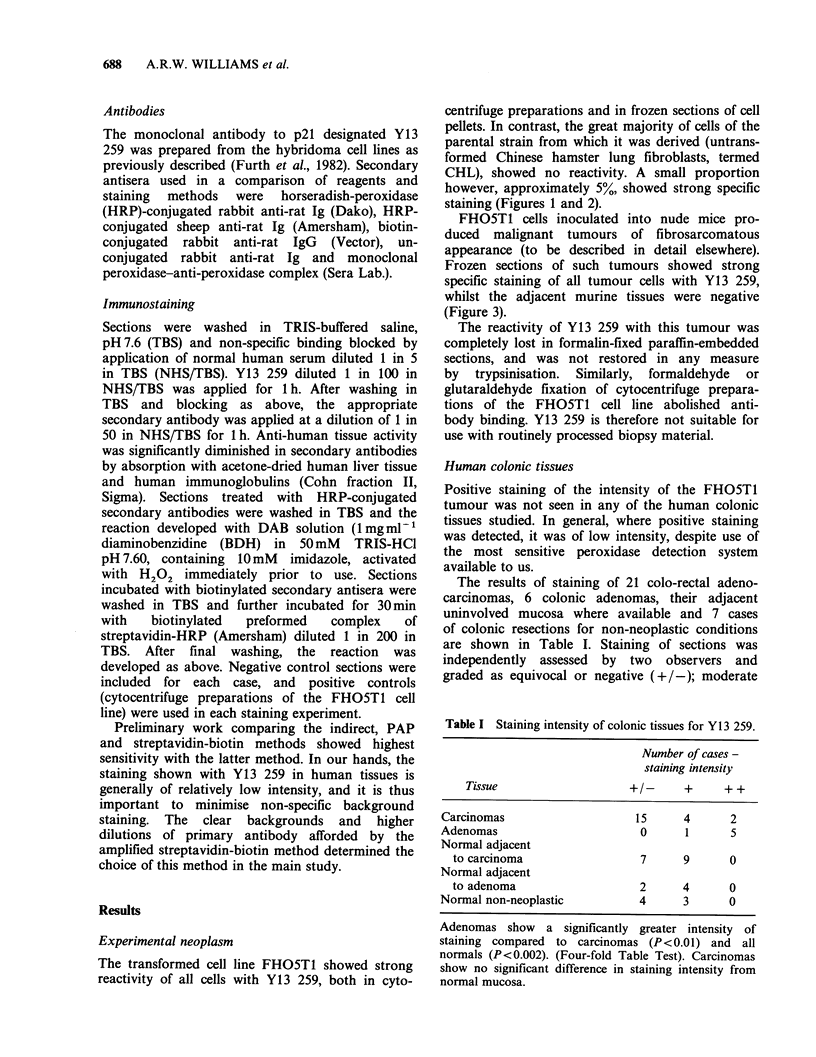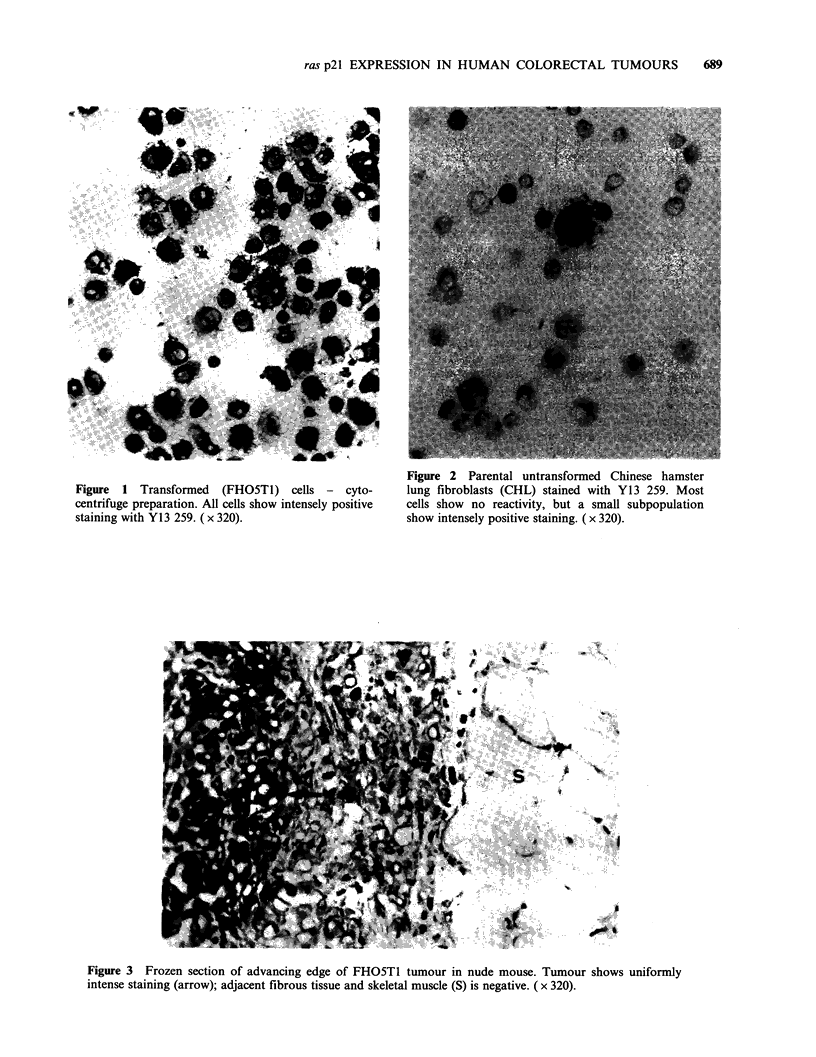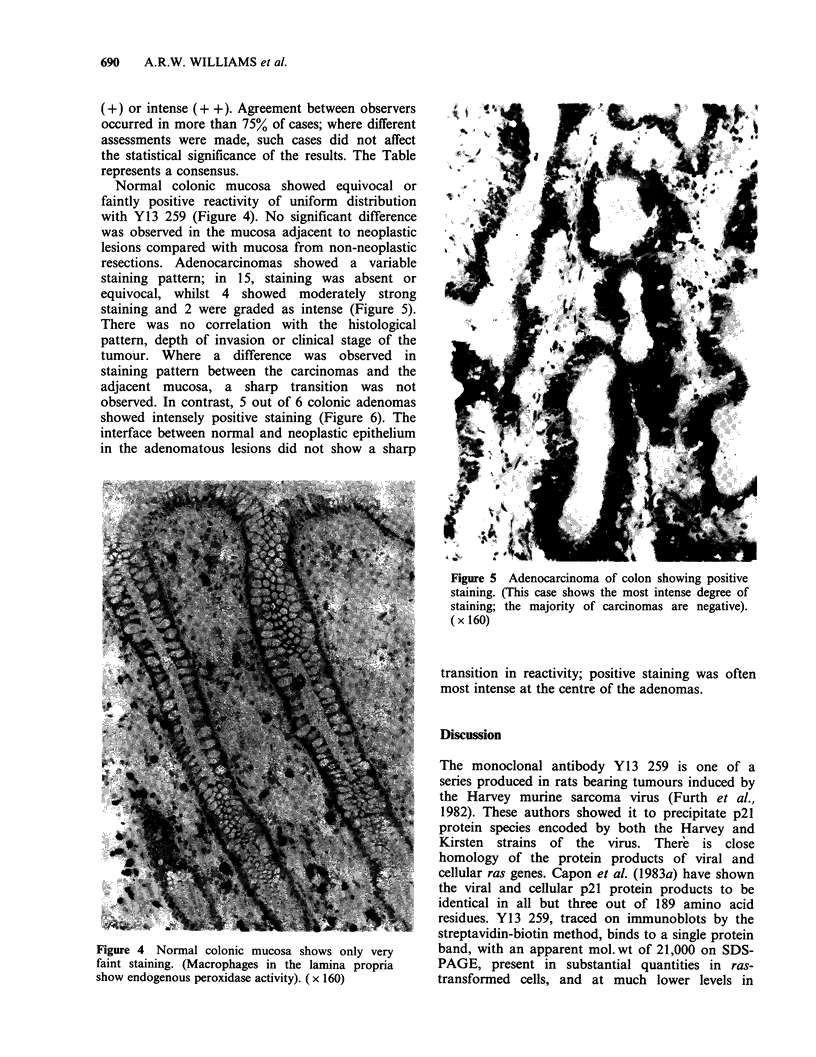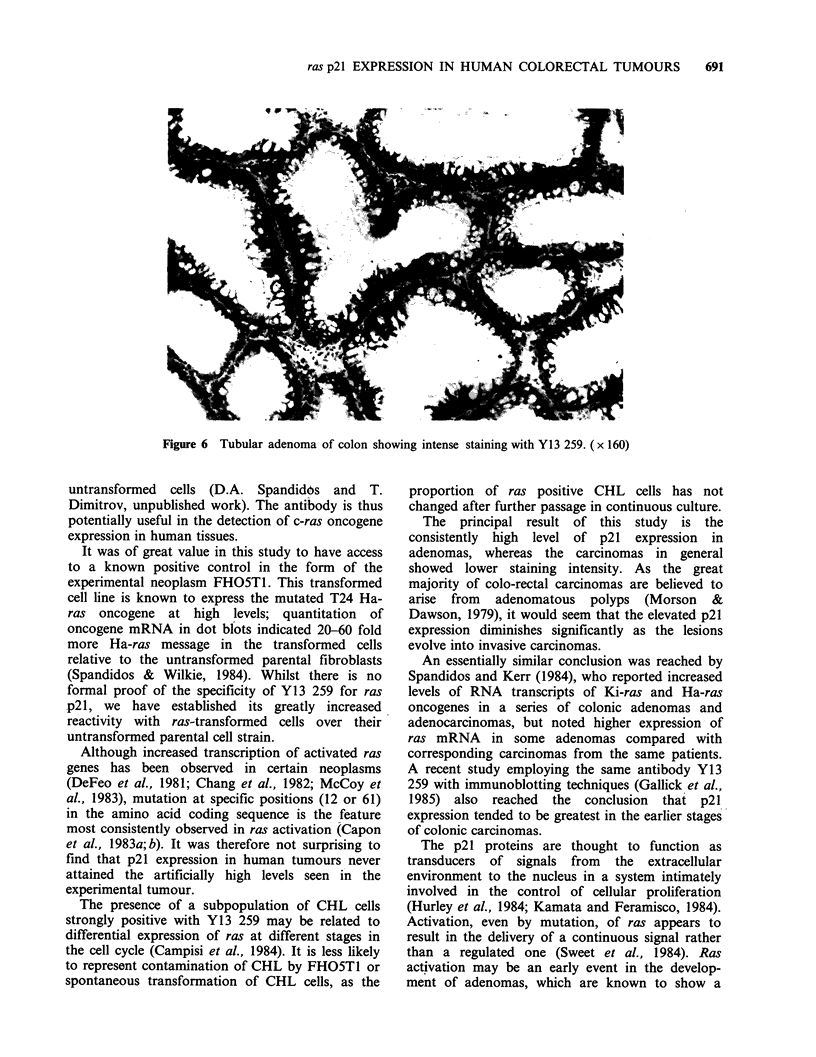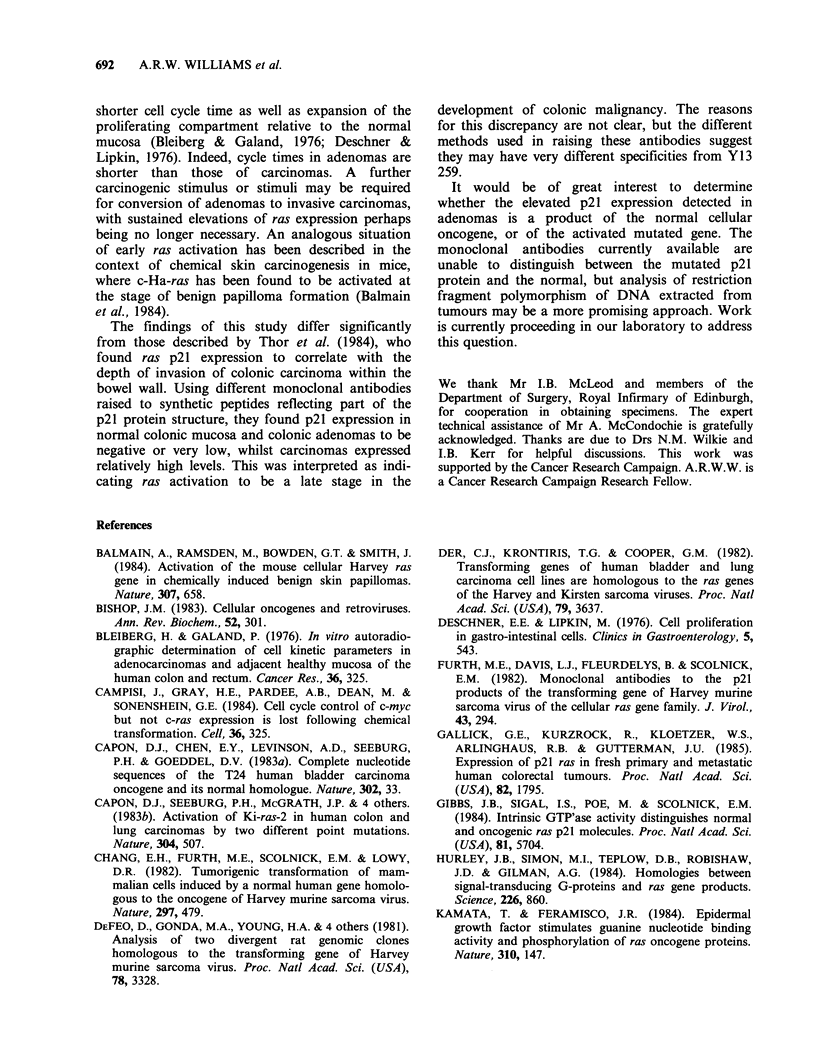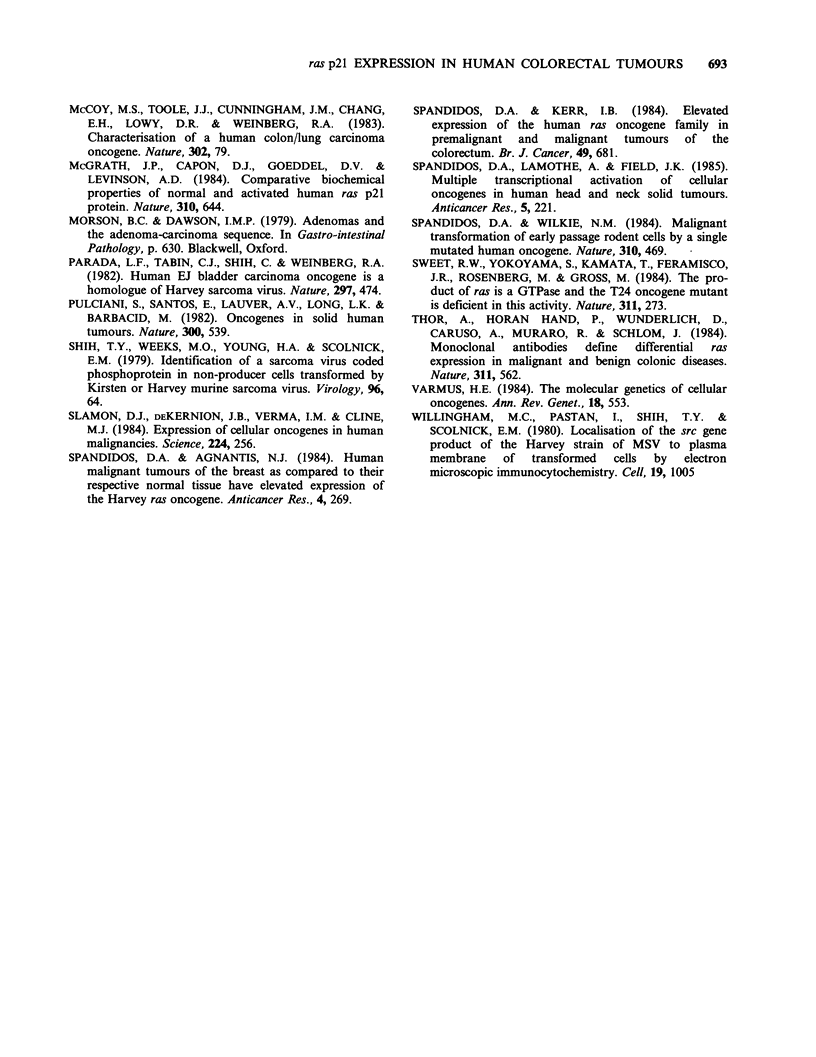# Immunohistochemical detection of the ras oncogene p21 product in an experimental tumour and in human colorectal neoplasms.

**DOI:** 10.1038/bjc.1985.244

**Published:** 1985-11

**Authors:** A. R. Williams, J. Piris, D. A. Spandidos, A. H. Wyllie

## Abstract

**Images:**


*Br. J. Cancer* (1985), 687–693

### Immunohistochemical detection of the *ras* oncogene p21 product in an experimental tumour and in human colorectal neoplasms

A.R.W. Williams[1], J. Piris[1], D.A. Spandidos[2,3] & A.H. Wyllie[1]

[1]*Department of Pathology, Edinburgh University Medical School, Teviot Place, Edinburgh EH8 9AG;* [2]*Beatson Institute for Cancer Research, Garscube Estate, Switchback Road, Bearsden, Glasgow G61 1BD, UK and* [3]*Hellenic Institut Pasteur, Athens, Greece.*

**Summary** The monoclonal antibody Y13 259 to the *ras* oncogene protein product p21 was used in an immunohistochemical study of *ras* expression in human colorectal neoplasms. The ability of the antibody to detect enhanced levels of *ras* expression was confirmed by its use with an experimental neoplasm known to express *ras* at high levels. Human colonic adenocarcinomas in general showed a similar staining intensity to that seen in normal mucosa. Adenomas however showed consistently high p21 expression as indicated by staining intensity. This suggests that elevated *ras* expression may be important in the development of adenomas, but that high levels need not be sustained in the conversion to invasive carcinoma.

Although the *ras* gene family has been studied longer than any other group of cellular oncogenes, there is still uncertainty over its role in authentic human carcinogenesis. *Ras* genes code for GTP-binding and GTP'ase enzyme activities of molecular weight 21,000 daltons, referred to as p21 (Shih *et al.*, 1979; Gibbs *et al.*, 1984; McGrath *et al.*, 1984; Sweet *et al.*, 1984), which appear to be located on the inner face of the plasma membrane (Willingham *et al.*, 1980), and are probably normally involved in the transduction of membrane-associated stimuli for cell proliferation (Bishop, 1983; Kamata & Feramisco, 1984). Activation of the genes by mutation has been detected in approximately 15% of a wide variety of spontaneous human tumours and transformed cell lines in culture (Der *et al.*, 1982; Parada *et al.*, 1982; Pulciani *et al.*, 1982; Varmus, 1984). Raised levels of *ras* transcript have also been identified in RNA extracted from several types of human tumour (Spandidos & Kerr, 1984; Slamon *et al.*, 1984; Spandidos & Agnantis, 1984; Spandidos *et al.*, 1985). Controversy remains, however, over whether *ras* activation is a primary event in carcinogenesis or appears during tumour progression. At a more pragmatic level, it is quite obscure whether the detection of *ras* expression in pre-malignant lesions or malignant tumours would provide useful diagnostic or prognostic information.

In this paper, we describe the use of the monoclonal antibody Y13 259 in detection of *ras* p21 in human colorectal neoplasms. We have confirmed the ability of the antibody to detect enhanced levels of *ras* expression by applying it in parallel to the experimental neoplasm FHO5T1, in which high levels of *ras* were achieved through genetic manipulation.

Correspondence: A.R.W. Williams.
Received 10 June 1985; and in revised form, 16 July 1985.

### Materials and methods

### *Tissues*

Tissues used included 21 adenocarcinomas of the colon and rectum, 6 benign tubular adenomas of the colon, and 7 specimens of colonic tissue resected for non-neoplastic conditions (diverticular disease or ulcerative colitis). The diagnosis in each case was confirmed on paraffin sections. Specimens were received fresh within minutes of resection in the operating theatre. They were examined immediately, samples of tissue taken (including where possible the tumour/normal interface), placed in plastic vials and 'snap' frozen in liquid nitrogen where they were stored until required. Frozen sections (6 $\mu$m) were cut, mounted on gelatine-coated slides, fixed in acetone at room temperature for 15 min and air dried. As controls for immuno-staining, two cell lines were maintained in culture as previously described (Spandidos & Wilkie, 1984): the transformed line FHO5T1, which contains the mutated T24 human Ha-*ras* oncogene inserted within a high expression vector, and its parental, untransformed Chinese hamster lung fibroblast strain, here called CHL. FHO5T1 cells were also grown as tumours in nude mice. Frozen sections of these tumours and normal mouse tissues were prepared exactly as above. The cultured cells were studied in cytocentrifuge preparations, or in frozen sections of pellets in low melting temperature agarose, fixed as above.

© The Macmillan Press Ltd., 1985

*Antibodies*

The monoclonal antibody to p21 designated Y13 259 was prepared from the hybridoma cell lines as previously described (Furth *et al.*, 1982). Secondary antisera used in a comparison of reagents and staining methods were horseradish-peroxidase (HRP)-conjugated rabbit anti-rat Ig (Dako), HRP-conjugated sheep anti-rat Ig (Amersham), biotin-conjugated rabbit anti-rat IgG (Vector), unconjugated rabbit anti-rat Ig and monoclonal peroxidase–anti-peroxidase complex (Sera Lab.).

*Immunostaining*

Sections were washed in TRIS-buffered saline, pH 7.6 (TBS) and non-specific binding blocked by application of normal human serum diluted 1 in 5 in TBS (NHS/TBS). Y13 259 diluted 1 in 100 in NHS/TBS was applied for 1 h. After washing in TBS and blocking as above, the appropriate secondary antibody was applied at a dilution of 1 in 50 in NHS/TBS for 1 h. Anti-human tissue activity was significantly diminished in secondary antibodies by absorption with acetone-dried human liver tissue and human immunoglobulins (Cohn fraction II, Sigma). Sections treated with HRP-conjugated secondary antibodies were washed in TBS and the reaction developed with DAB solution ($1\,mg\,ml^{-1}$ diaminobenzidine (BDH) in 50 mM TRIS-HCl pH 7.60, containing 10 mM imidazole, activated with $H_2O_2$ immediately prior to use. Sections incubated with biotinylated secondary antisera were washed in TBS and further incubated for 30 min with biotinylated preformed complex of streptavidin-HRP (Amersham) diluted 1 in 200 in TBS. After final washing, the reaction was developed as above. Negative control sections were included for each case, and positive controls (cytocentrifuge preparations of the FHO5T1 cell line) were used in each staining experiment.

Preliminary work comparing the indirect, PAP and streptavidin-biotin methods showed highest sensitivity with the latter method. In our hands, the staining shown with Y13 259 in human tissues is generally of relatively low intensity, and it is thus important to minimise non-specific background staining. The clear backgrounds and higher dilutions of primary antibody afforded by the amplified streptavidin-biotin method determined the choice of this method in the main study.

### Results

*Experimental neoplasm*

The transformed cell line FHO5T1 showed strong reactivity of all cells with Y13 259, both in cytocentrifuge preparations and in frozen sections of cell pellets. In contrast, the great majority of cells of the parental strain from which it was derived (untransformed Chinese hamster lung fibroblasts, termed CHL), showed no reactivity. A small proportion however, approximately 5%, showed strong specific staining (Figures 1 and 2).

FHO5T1 cells inoculated into nude mice produced malignant tumours of fibrosarcomatous appearance (to be described in detail elsewhere). Frozen sections of such tumours showed strong specific staining of all tumour cells with Y13 259, whilst the adjacent murine tissues were negative (Figure 3).

The reactivity of Y13 259 with this tumour was completely lost in formalin-fixed paraffin-embedded sections, and was not restored in any measure by trypsinisation. Similarly, formaldehyde or glutaraldehyde fixation of cytocentrifuge preparations of the FHO5T1 cell line abolished antibody binding. Y13 259 is therefore not suitable for use with routinely processed biopsy material.

*Human colonic tissues*

Positive staining of the intensity of the FHO5T1 tumour was not seen in any of the human colonic tissues studied. In general, where positive staining was detected, it was of low intensity, despite use of the most sensitive peroxidase detection system available to us.

The results of staining of 21 colo-rectal adenocarcinomas, 6 colonic adenomas, their adjacent uninvolved mucosa where available and 7 cases of colonic resections for non-neoplastic conditions are shown in Table I. Staining of sections was independently assessed by two observers and graded as equivocal or negative (+/−); moderate

**Table I** Staining intensity of colonic tissues for Y13 259.

| Tissue | *Number of cases – staining intensity* +/− | + | ++ |
|---|---|---|---|
| Carcinomas | 15 | 4 | 2 |
| Adenomas | 0 | 1 | 5 |
| Normal adjacent to carcinoma | 7 | 9 | 0 |
| Normal adjacent to adenoma | 2 | 4 | 0 |
| Normal non-neoplastic | 4 | 3 | 0 |

Adenomas show a significantly greater intensity of staining compared to carcinomas ($P<0.01$) and all normals ($P<0.002$). (Four-fold Table Test). Carcinomas show no significant difference in staining intensity from normal mucosa.

**Figure 1** Transformed (FHO5T1) cells – cytocentrifuge preparation. All cells show intensely positive staining with Y13 259. ( × 320).

**Figure 2** Parental untransformed Chinese hamster lung fibroblasts (CHL) stained with Y13 259. Most cells show no reactivity, but a small subpopulation show intensely positive staining. ( × 320).

**Figure 3** Frozen section of advancing edge of FHO5T1 tumour in nude mouse. Tumour shows uniformly intense staining (arrow); adjacent fibrous tissue and skeletal muscle (S) is negative. ( × 320).

(+) or intense (+ +). Agreement between observers occurred in more than 75% of cases; where different assessments were made, such cases did not affect the statistical significance of the results. The Table represents a consensus.

Normal colonic mucosa showed equivocal or faintly positive reactivity of uniform distribution with Y13 259 (Figure 4). No significant difference was observed in the mucosa adjacent to neoplastic lesions compared with mucosa from non-neoplastic resections. Adenocarcinomas showed a variable staining pattern; in 15, staining was absent or equivocal, whilst 4 showed moderately strong staining and 2 were graded as intense (Figure 5). There was no correlation with the histological pattern, depth of invasion or clinical stage of the tumour. Where a difference was observed in staining pattern between the carcinomas and the adjacent mucosa, a sharp transition was not observed. In contrast, 5 out of 6 colonic adenomas showed intensely positive staining (Figure 6). The interface between normal and neoplastic epithelium in the adenomatous lesions did not show a sharp transition in reactivity; positive staining was often most intense at the centre of the adenomas.

**Figure 4** Normal colonic mucosa shows only very faint staining. (Macrophages in the lamina propria show endogenous peroxidase activity). (×160)

**Figure 5** Adenocarcinoma of colon showing positive staining. (This case shows the most intense degree of staining; the majority of carcinomas are negative). (×160)

### Discussion

The monoclonal antibody Y13 259 is one of a series produced in rats bearing tumours induced by the Harvey murine sarcoma virus (Furth *et al.*, 1982). These authors showed it to precipitate p21 protein species encoded by both the Harvey and Kirsten strains of the virus. There is close homology of the protein products of viral and cellular *ras* genes. Capon *et al.* (1983*a*) have shown the viral and cellular p21 protein products to be identical in all but three out of 189 amino acid residues. Y13 259, traced on immunoblots by the streptavidin-biotin method, binds to a single protein band, with an apparent mol. wt of 21,000 on SDS-PAGE, present in substantial quantities in *ras*-transformed cells, and at much lower levels in

**Figure 6** Tubular adenoma of colon showing intense staining with Y13 259. (×160)

untransformed cells (D.A. Spandidos and T. Dimitrov, unpublished work). The antibody is thus potentially useful in the detection of c-*ras* oncogene expression in human tissues.

It was of great value in this study to have access to a known positive control in the form of the experimental neoplasm FHO5T1. This transformed cell line is known to express the mutated T24 Ha-*ras* oncogene at high levels; quantitation of oncogene mRNA in dot blots indicated 20–60 fold more Ha-*ras* message in the transformed cells relative to the untransformed parental fibroblasts (Spandidos & Wilkie, 1984). Whilst there is no formal proof of the specificity of Y13 259 for *ras* p21, we have established its greatly increased reactivity with *ras*-transformed cells over their untransformed parental cell strain.

Although increased transcription of activated *ras* genes has been observed in certain neoplasms (DeFeo *et al.*, 1981; Chang *et al.*, 1982; McCoy *et al.*, 1983), mutation at specific positions (12 or 61) in the amino acid coding sequence is the feature most consistently observed in *ras* activation (Capon *et al.*, 1983*a*; *b*). It was therefore not surprising to find that p21 expression in human tumours never attained the artificially high levels seen in the experimental tumour.

The presence of a subpopulation of CHL cells strongly positive with Y13 259 may be related to differential expression of *ras* at different stages in the cell cycle (Campisi *et al.*, 1984). It is less likely to represent contamination of CHL by FHO5T1 or spontaneous transformation of CHL cells, as the proportion of *ras* positive CHL cells has not changed after further passage in continuous culture.

The principal result of this study is the consistently high level of p21 expression in adenomas, whereas the carcinomas in general showed lower staining intensity. As the great majority of colo-rectal carcinomas are believed to arise from adenomatous polyps (Morson & Dawson, 1979), it would seem that the elevated p21 expression diminishes significantly as the lesions evolve into invasive carcinomas.

An essentially similar conclusion was reached by Spandidos and Kerr (1984), who reported increased levels of RNA transcripts of Ki-*ras* and Ha-*ras* oncogenes in a series of colonic adenomas and adenocarcinomas, but noted higher expression of *ras* mRNA in some adenomas compared with corresponding carcinomas from the same patients. A recent study employing the same antibody Y13 259 with immunoblotting techniques (Gallick *et al.*, 1985) also reached the conclusion that p21 expression tended to be greatest in the earlier stages of colonic carcinomas.

The p21 proteins are thought to function as transducers of signals from the extracellular environment to the nucleus in a system intimately involved in the control of cellular proliferation (Hurley *et al.*, 1984; Kamata and Feramisco, 1984). Activation, even by mutation, of *ras* appears to result in the delivery of a continuous signal rather than a regulated one (Sweet *et al.*, 1984). *Ras* activation may be an early event in the development of adenomas, which are known to show a shorter cell cycle time as well as expansion of the proliferating compartment relative to the normal mucosa (Bleiberg & Galand, 1976; Deschner & Lipkin, 1976). Indeed, cycle times in adenomas are shorter than those of carcinomas. A further carcinogenic stimulus or stimuli may be required for conversion of adenomas to invasive carcinomas, with sustained elevations of *ras* expression perhaps being no longer necessary. An analogous situation of early *ras* activation has been described in the context of chemical skin carcinogenesis in mice, where c-Ha-*ras* has been found to be activated at the stage of benign papilloma formation (Balmain *et al.*, 1984).

The findings of this study differ significantly from those described by Thor *et al.* (1984), who found *ras* p21 expression to correlate with the depth of invasion of colonic carcinoma within the bowel wall. Using different monoclonal antibodies raised to synthetic peptides reflecting part of the p21 protein structure, they found p21 expression in normal colonic mucosa and colonic adenomas to be negative or very low, whilst carcinomas expressed relatively high levels. This was interpreted as indicating *ras* activation to be a late stage in the development of colonic malignancy. The reasons for this discrepancy are not clear, but the different methods used in raising these antibodies suggest they may have very different specificities from Y13 259.

It would be of great interest to determine whether the elevated p21 expression detected in adenomas is a product of the normal cellular oncogene, or of the activated mutated gene. The monoclonal antibodies currently available are unable to distinguish between the mutated p21 protein and the normal, but analysis of restriction fragment polymorphism of DNA extracted from tumours may be a more promising approach. Work is currently proceeding in our laboratory to address this question.

We thank Mr I.B. McLeod and members of the Department of Surgery, Royal Infirmary of Edinburgh, for cooperation in obtaining specimens. The expert technical assistance of Mr A. McCondochie is gratefully acknowledged. Thanks are due to Drs N.M. Wilkie and I.B. Kerr for helpful discussions. This work was supported by the Cancer Research Campaign. A.R.W.W. is a Cancer Research Campaign Research Fellow.

### References

BALMAIN, A., RAMSDEN, M., BOWDEN, G.T. & SMITH, J. (1984). Activation of the mouse cellular Harvey *ras* gene in chemically induced benign skin papillomas. *Nature*, **307**, 658.

BISHOP, J.M. (1983). Cellular oncogenes and retroviruses. *Ann. Rev. Biochem.*, **52**, 301.

BLEIBERG, H. & GALAND, P. (1976). *In vitro* autoradiographic determination of cell kinetic parameters in adenocarcinomas and adjacent healthy mucosa of the human colon and rectum. *Cancer Res.*, **36**, 325.

CAMPISI, J., GRAY, H.E., PARDEE, A.B., DEAN, M. & SONENSHEIN, G.E. (1984). Cell cycle control of c-*myc* but not c-*ras* expression is lost following chemical transformation. *Cell*, **36**, 325.

CAPON, D.J., CHEN, E.Y., LEVINSON, A.D., SEEBURG, P.H. & GOEDDEL, D.V. (1983*a*). Complete nucleotide sequences of the T24 human bladder carcinoma oncogene and its normal homologue. *Nature*, **302**, 33.

CAPON, D.J., SEEBURG, P.H., McGRATH, J.P. & 4 others. (1983*b*). Activation of Ki-*ras*-2 in human colon and lung carcinomas by two different point mutations. *Nature*, **304**, 507.

CHANG, E.H., FURTH, M.E., SCOLNICK, E.M. & LOWY, D.R. (1982). Tumorigenic transformation of mammalian cells induced by a normal human gene homologous to the oncogene of Harvey murine sarcoma virus. *Nature*, **297**, 479.

DEFEO, D., GONDA, M.A., YOUNG, H.A. & 4 others (1981). Analysis of two divergent rat genomic clones homologous to the transforming gene of Harvey murine sarcoma virus. *Proc. Natl Acad. Sci.* (*USA*), **78**, 3328.

DER, C.J., KRONTIRIS, T.G. & COOPER, G.M. (1982). Transforming genes of human bladder and lung carcinoma cell lines are homologous to the *ras* genes of the Harvey and Kirsten sarcoma viruses. *Proc. Natl Acad. Sci.* (*USA*), **79**, 3637.

DESCHNER, E.E. & LIPKIN, M. (1976). Cell proliferation in gastro-intestinal cells. *Clinics in Gastroenterology*, **5**, 543.

FURTH, M.E., DAVIS, L.J., FLEURDELYS, B. & SCOLNICK, E.M. (1982). Monoclonal antibodies to the p21 products of the transforming gene of Harvey murine sarcoma virus of the cellular *ras* gene family. *J. Virol.*, **43**, 294.

GALLICK, G.E., KURZROCK, R., KLOETZER, W.S., ARLINGHAUS, R.B. & GUTTERMAN, J.U. (1985). Expression of p21 *ras* in fresh primary and metastatic human colorectal tumours. *Proc. Natl Acad. Sci.* (*USA*), **82**, 1795.

GIBBS, J.B., SIGAL, I.S., POE, M. & SCOLNICK, E.M. (1984). Intrinsic GTP'ase activity distinguishes normal and oncogenic *ras* p21 molecules. *Proc. Natl Acad. Sci.* (*USA*), **81**, 5704.

HURLEY, J.B., SIMON, M.I., TEPLOW, D.B., ROBISHAW, J.D. & GILMAN, A.G. (1984). Homologies between signal-transducing G-proteins and *ras* gene products. *Science*, **226**, 860.

KAMATA, T. & FERAMISCO, J.R. (1984). Epidermal growth factor stimulates guanine nucleotide binding activity and phosphorylation of *ras* oncogene proteins. *Nature*, **310**, 147.

McCOY, M.S., TOOLE, J.J., CUNNINGHAM, J.M., CHANG, E.H., LOWY, D.R. & WEINBERG, R.A. (1983). Characterisation of a human colon/lung carcinoma oncogene. *Nature*, **302**, 79.

McGRATH, J.P., CAPON, D.J., GOEDDEL, D.V. & LEVINSON, A.D. (1984). Comparative biochemical properties of normal and activated human *ras* p21 protein. *Nature*, **310**, 644.

MORSON, B.C. & DAWSON, I.M.P. (1979). Adenomas and the adenoma-carcinoma sequence. In *Gastro-intestinal Pathology*, p. 630. Blackwell, Oxford.

PARADA, L.F., TABIN, C.J., SHIH, C. & WEINBERG, R.A. (1982). Human EJ bladder carcinoma oncogene is a homologue of Harvey sarcoma virus. *Nature*, **297**, 474.

PULCIANI, S., SANTOS, E., LAUVER, A.V., LONG, L.K. & BARBACID, M. (1982). Oncogenes in solid human tumours. *Nature*, **300**, 539.

SHIH, T.Y., WEEKS, M.O., YOUNG, H.A. & SCOLNICK, E.M. (1979). Identification of a sarcoma virus coded phosphoprotein in non-producer cells transformed by Kirsten or Harvey murine sarcoma virus. *Virology*, **96**, 64.

SLAMON, D.J., DEKERNION, J.B., VERMA, I.M. & CLINE, M.J. (1984). Expression of cellular oncogenes in human malignancies. *Science*, **224**, 256.

SPANDIDOS, D.A. & AGNANTIS, N.J. (1984). Human malignant tumours of the breast as compared to their respective normal tissue have elevated expression of the Harvey *ras* oncogene. *Anticancer Res.*, **4**, 269.

SPANDIDOS, D.A. & KERR, I.B. (1984). Elevated expression of the human *ras* oncogene family in premalignant and malignant tumours of the colorectum. *Br. J. Cancer*, **49**, 681.

SPANDIDOS, D.A., LAMOTHE, A. & FIELD, J.K. (1985). Multiple transcriptional activation of cellular oncogenes in human head and neck solid tumours. *Anticancer Res.*, **5**, 221.

SPANDIDOS, D.A. & WILKIE, N.M. (1984). Malignant transformation of early passage rodent cells by a single mutated human oncogene. *Nature*, **310**, 469.

SWEET, R.W., YOKOYAMA, S., KAMATA, T., FERAMISCO, J.R., ROSENBERG, M. & GROSS, M. (1984). The product of *ras* is a GTPase and the T24 oncogene mutant is deficient in this activity. *Nature*, **311**, 273.

THOR, A., HORAN HAND, P., WUNDERLICH, D., CARUSO, A., MURARO, R. & SCHLOM, J. (1984). Monoclonal antibodies define differential *ras* expression in malignant and benign colonic diseases. *Nature*, **311**, 562.

VARMUS, H.E. (1984). The molecular genetics of cellular oncogenes. *Ann. Rev. Genet.*, **18**, 553.

WILLINGHAM, M.C., PASTAN, I., SHIH, T.Y. & SCOLNICK, E.M. (1980). Localisation of the *src* gene product of the Harvey strain of MSV to plasma membrane of transformed cells by electron microscopic immunocytochemistry. *Cell*, **19**, 1005